# Potential Therapeutic Synergy of High-Dose Vitamin C and Nicotinamide as Adjunctive Therapy in an Elderly Patient With Extensive-Stage Small-Cell Lung Cancer: A Case Report

**DOI:** 10.7759/cureus.101050

**Published:** 2026-01-07

**Authors:** Minsu Lee, Wan-ki Park, Jae-Hyeok Lee, Won-Seok Kang, Je H Jeong

**Affiliations:** 1 Department of Advanced Bio-Convergence, Chungbuk Health and Science University, Cheongju-si, KOR; 2 Center for Bio-Signal Research, Korea Institute of Toxicology, Daejeon , KOR; 3 Department of Molecular Science and Technology, Ajou University, Suwon, KOR; 4 Department of Neurological Surgery, Hangang Sacred Heart Hospital, College of Medicine, Hallym University, Seoul, KOR

**Keywords:** chemoimmunotherapy, elderly patients, extensive-stage, large mediastinal mass, megadose, metabolic adjunctive therapy, nicotinamide (vitamin b3), oral administration, small-cell lung cancer (sclc), vitamin c

## Abstract

We report the case of a 78-year-old male with extensive-stage small-cell lung cancer (ES-SCLC) involving the brain, bone, and adrenal gland at diagnosis, who exhibited a near-complete thoracic radiologic response following platinum-etoposide-atezolizumab therapy with consolidative thoracic and whole-brain radiotherapy. In addition to standard chemoimmunotherapy, the patient self-administered high-dose oral vitamin C (3g/day) and nicotinamide (3g/day) continuously throughout treatment, raising the possibility that metabolic adjunctive therapy may have contributed to the depth of response. Initial CT and fluoro-ethyl-tyrosine (FET) fusion imaging showed a large mediastinal mass, which nearly disappeared after five cycles of treatment. This case highlights that substantial therapeutic responses are achievable even in elderly patients with extensive metastatic disease and suggests that adjunctive metabolic modulation with high-dose vitamin supplementation may warrant further investigation as a potential enhancer of response to chemoimmunotherapy.

## Introduction

Small-cell lung cancer (SCLC) is a highly aggressive neuroendocrine carcinoma characterized by rapid doubling time, early hematogenous metastasis, and high initial chemosensitivity [1]. Approximately two-thirds of patients present with extensive-stage disease, defined by metastatic spread beyond a single tolerable radiation field [1]. Diagnosis typically relies on cross-sectional imaging and histopathological confirmation via bronchoscopic or needle biopsy, and staging evaluation often includes brain magnetic resonance imaging (MRI) and positron emission tomography/computed tomography (PET/CT) [1-3].

The current standard first-line therapy for extensive-stage SCLC (ES-SCLC) consists of platinum-etoposide chemotherapy combined with immunotherapy using programmed death-ligand 1 (PD-L1) inhibitors such as atezolizumab or durvalumab [3-5]. These regimens modestly improve median overall survival to approximately 12-13 months [4,5]. In selected patients, consolidative thoracic radiotherapy and cranial irradiation may further enhance local control and delay progression [3,6].

Beyond conventional modalities, there is growing interest in metabolic adjunctive therapies that may potentiate antitumor effects when combined with chemoimmunotherapy. High-dose vitamin C (ascorbate) has been reported in preclinical and early clinical studies to exert selective cytotoxicity in cancer cells through hydrogen peroxide generation, disruption of redox homeostasis, and enhancement of DNA damage induced by chemotherapy and radiotherapy, as well as to modulate the tumor microenvironment and immune responses. However, it should be emphasized that the mechanistic effects of high-dose vitamin C described in preclinical models and early-phase clinical studies do not establish a causal relationship with clinical tumor response in individual patients. In the context of a single case report, such observations should be interpreted cautiously and regarded as hypothesis-generating rather than confirmatory. 

However, it should be emphasized that the mechanistic effects of high-dose vitamin C described in preclinical models and early-phase clinical studies do not establish a causal relationship with clinical tumor response in individual patients. In the context of a single case report, such observations should be interpreted cautiously and regarded as hypothesis-generating rather than confirmatory.

Nicotinamide, a form of vitamin B3 and a precursor in the NAD+ salvage pathway, has gained attention for its role in DNA repair modulation, epigenetic regulation, and tumor metabolism [7,8]. Nicotinamide is an inhibitor of human sirtuin (HDAC III) and is known to reactivate RUNX family transcription factor 3 (RUNX3), a tumor suppressor that is epigenetically suppressed in cancer cells. Nicotinamide is a component that has been confirmed to be effective in various animal cancer models, including lung cancer, bladder cancer, and liver cancer [9-11]. A 2024 phase IIb randomized clinical trial in epidermal growth factor receptor (EGFR)-mutant lung adenocarcinoma demonstrated that nicotinamide may augment responses to EGFR tyrosine kinase inhibitors, supporting its biologic plausibility as an adjunct to systemic anticancer treatment [12]. It should be noted that the majority of available clinical evidence supporting nicotinamide as an adjunctive anticancer agent is derived from preclinical studies or clinical trials conducted in non-small cell lung cancer. Given the distinct biology and treatment responsiveness of small-cell lung cancer, direct extrapolation of these findings to ES-SCLC is not possible, and such data are cited here solely to support biological plausibility rather than therapeutic equivalence.

It should be noted that the majority of available clinical evidence supporting nicotinamide as an adjunctive anticancer agent is derived from preclinical studies or clinical trials conducted in non-small cell lung cancer. Given the distinct biology and treatment responsiveness of small-cell lung cancer, direct extrapolation of these findings to ES-SCLC is not possible, and such data are cited here solely to support biological plausibility rather than therapeutic equivalence.

Recent case reports have described rare long-term survivors achieving multi-year remission after platinum-etoposide-PD-L1 inhibitor therapy, sometimes augmented by thoracic radiotherapy; however, such outcomes remain uncommon, particularly in elderly patients with extensive metastases [13-15]. Here, we report a 78-year-old ES-SCLC patient who received standard chemoimmunotherapy and consolidative radiation while concurrently self-administering high-dose vitamin C and nicotinamide. The patient demonstrated substantial radiologic tumor regression, documented through comparative CT and FET imaging. This case provides an opportunity to explore the hypothesis-generating potential of metabolic adjunctive therapy as a contributor to the depth of clinical response. Accordingly, this report does not seek to establish a causal effect of metabolic adjunctive therapy, but rather to describe an uncommon depth of radiologic response in an elderly patient with ES-SCLC and to raise a hypothesis that may warrant further investigation.

## Case presentation

Methods

Patient Information and Clinical Background

The patient was a 78-year-old male with a history of type 2 diabetes mellitus (>10 years), coronary artery stent placement (×2), and long-term smoking, with a family history of lung cancer. He presented with progressive dyspnea and cough in May 2025. Imaging and pathological evaluation confirmed extensive-stage small-cell lung cancer with metastases to the brain, bone, and adrenal gland.

Diagnostic Evaluation

Immunohistochemical analysis of the biopsy specimen revealed tumor cells positive for neuroendocrine markers, including synaptophysin and chromogranin A, with strong nuclear expression of thyroid transcription factor-1 (TTF-1). The tumor cells were negative for markers of non-small cell lung carcinoma. These findings, together with the characteristic morphologic features, were consistent with a diagnosis of small-cell lung cancer.

Initial clinical evaluation included assessment of presenting respiratory symptoms, physical examination, and review of the patient’s smoking history. A contrast-enhanced CT scan was performed to evaluate the size and anatomical extent of the primary tumor. Histopathologic confirmation was obtained via bronchoscopic biopsy of the left paratracheal mass. Contrast-enhanced brain MRI was conducted to assess for intracranial metastases, given the high propensity of SCLC for early brain involvement. Baseline assessment included chest CT and PET (fusion PET/CT). CT revealed an approximately 8.5 cm mediastinal mass extending from the upper esophagus to the anterior cardiac border, with compression of mediastinal structures. PET fusion imaging demonstrated intense metabolic activity at the lesion site, consistent with high-grade neuroendocrine carcinoma [2,16].

Radiological Comparison Analysis

**Figure 1:**
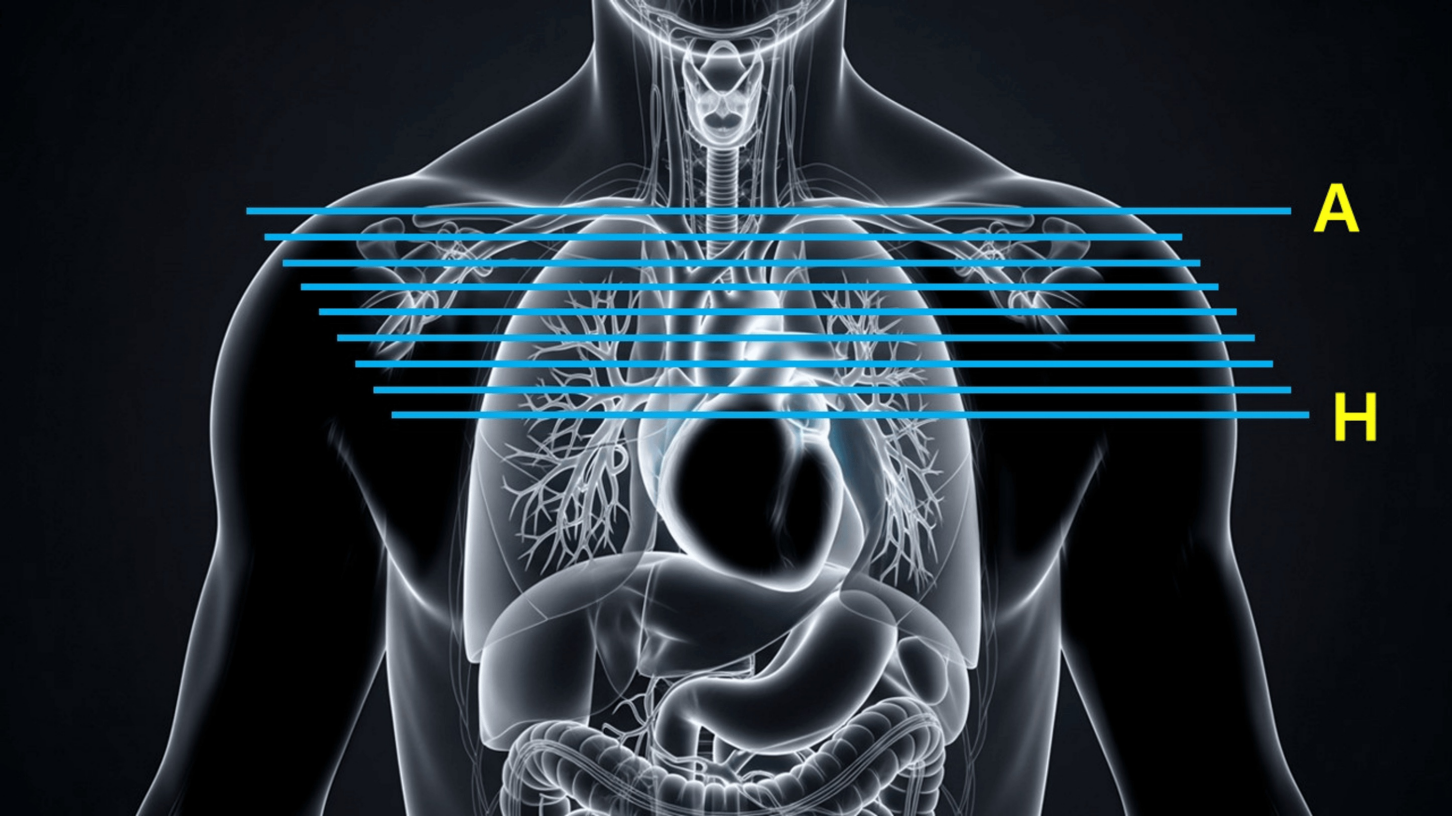
Anatomical correlation map for CT slice levels (A–H) Schematic anterior thoracic illustration displaying the approximate axial slice positions (A–H) used for comparative CT analysis. Slice A corresponds to the supraclavicular inlet, and slice H corresponds to the level of the cardiac ventricles. The image has been created by the authors.

Serial CT scans from June 5, 2025 (baseline) and October 9, 2025 (post-cycle 5) were reviewed. PET fusion imaging at baseline was compared with corresponding CT slices to assess metabolic activity. As shown in Figure 1, axial slices from the thoracic inlet to the cardiac upper border were aligned for direct comparison between time points. Radiologic tumor response was additionally assessed retrospectively according to the Response Evaluation Criteria in Solid Tumors (RECIST) guideline, version 1.1. A dominant measurable mediastinal mass identified on baseline contrast-enhanced CT was selected as the target lesion, and its longest diameter was evaluated on anatomically matched axial slices at baseline and follow-up imaging (Figure 1). This diagram provides anatomical reference for interpreting the serial CT images shown later in the article.

Treatment Approach

First-line systemic therapy consisted of atezolizumab, etoposide, and cisplatin administered in 21-day cycles. Combination therapy was initiated on June 10, 2025, and four cycles were completed through mid-August 2025, followed by maintenance atezolizumab beginning in early September 2025. Baseline radiologic evaluation with contrast-enhanced CT and PET/CT was performed on June 5, 2025, prior to initiation of systemic therapy, and follow-up CT imaging for response assessment was conducted on October 9, 2025, after completion of five cycles of systemic treatment.

Throughout the entire treatment period, the patient continuously self-administered high-dose oral vitamin C (3g/day) and nicotinamide (3g/day). No treatment interruption, dose modification, or clinically significant adverse events attributable to these supplements were observed. Consolidative thoracic radiotherapy and whole-brain radiotherapy were subsequently administered to the mediastinal primary region and brain metastases, respectively, in accordance with institutional practice and published evidence supporting the role of thoracic and cranial irradiation in ES-SCLC [3,6].

Results

Baseline Radiologic Findings

**Figure 2:**
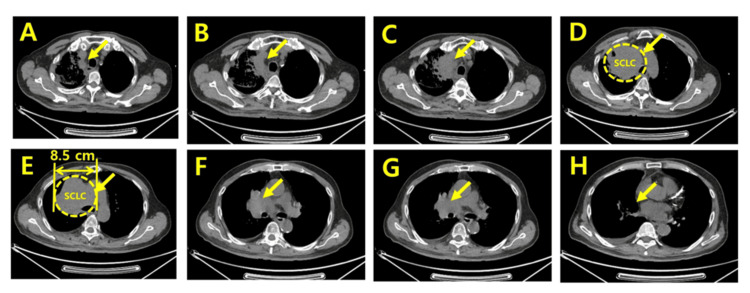
Baseline axial CT images captured immediately after diagnosis (2025-06-05, Slices A–H) A series of eight axial CT slices (A–H) from the thoracic inlet to the cardiac level obtained at initial diagnosis. Slices A–D reveal a large, irregular anterior mediastinal and right paratracheal mass with heterogeneous internal density and compression of adjacent mediastinal structures. Slices E–H show inferior extension of the mass with continued displacement of the mediastinum and partial effacement of the pericardial border. These images represent the full cranio-caudal extent of the primary tumor burden before systemic therapy. Arrows indicate small cell lung cancer sites.

Baseline CT imaging on June 5, 2025, revealed a large anterior mediastinal mass measuring approximately 8.5 cm, extending from the thoracic inlet to the upper cardiac border, with displacement and compression of adjacent mediastinal structures. Corresponding PET/CT demonstrated intense radiotracer uptake throughout the lesion, indicating a highly metabolically active, high-grade neuroendocrine carcinoma consistent with extensive-stage SCLC [2,16]. These findings reflected a substantial tumor burden in an elderly patient with multi-organ metastases (Figure 2).

Post-treatment Radiologic Response

**Figure 3:**
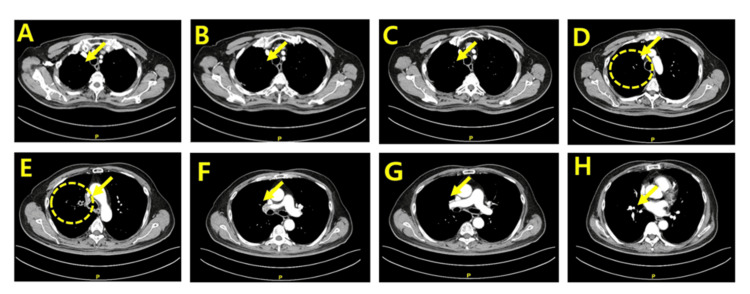
Post-treatment axial CT images after five cycles of therapy (2025-10-09, Slices A–H) Matched axial CT slices (A–H) obtained after completion of five cycles of platinum–etoposide–atezolizumab therapy. Across all levels, the previously identified mediastinal mass has regressed almost completely, with restoration of normal mediastinal contour, re-expansion of adjacent lung parenchyma, and absence of any mass-like density. Residual fibrosis is minimal, and no new intrathoracic lesions are detected. These images demonstrate a near-complete structural response to systemic therapy. Arrows indicate the site of resected small cell lung cancer.

As shown in Figure 3, following five cycles of platinum-etoposide-atezolizumab therapy, together with consolidative thoracic and whole-brain radiotherapy and concurrent high-dose vitamin C and nicotinamide supplementation, the CT scan dated October 9, 2025, demonstrated a striking near-complete radiologic response. Based on the RECIST, version 1.1-guided retrospective assessment, the selected target lesion demonstrated near-complete disappearance, with no residual measurable soft-tissue lesion identifiable at corresponding axial levels. Although precise serial measurements were limited by the retrospective nature of this analysis, the observed reduction in tumor burden exceeded the threshold for partial response and approached the criteria for complete response. Across all axial slice levels from the thoracic inlet to the cardiac upper border, the previously bulky mediastinal mass had nearly completely regressed, with restoration of normal mediastinal contours and improved aeration of the adjacent lung parenchyma. No new intrathoracic lesions or lymphadenopathy were identified (Figure 3). During the course of combination chemoimmunotherapy and subsequent maintenance atezolizumab, the patient did not experience any grade ≥3 adverse events or clinically meaningful toxicities attributable to concurrent high-dose vitamin C or nicotinamide supplementation.

**Figure 4:**
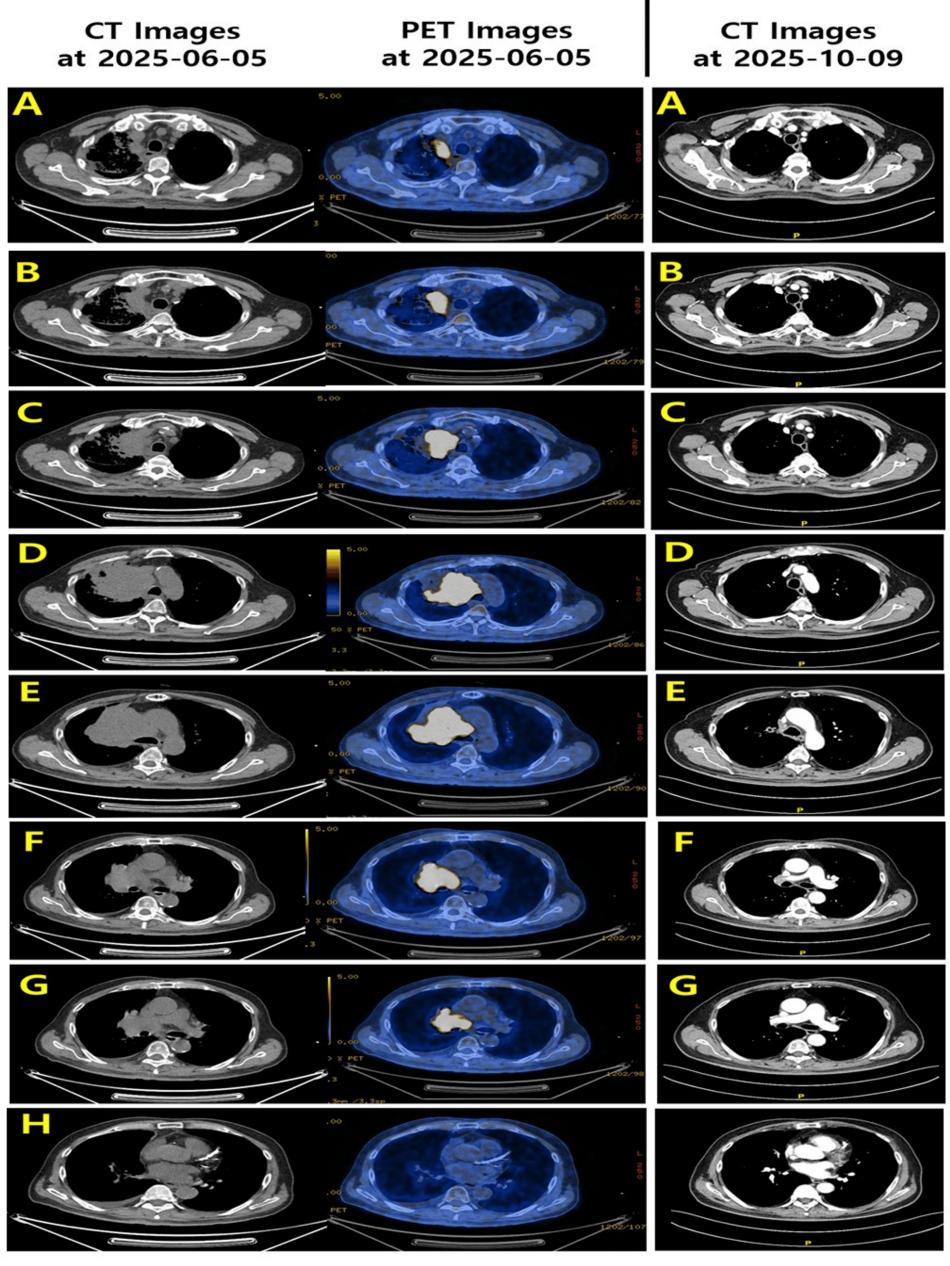
Serial axial CT and PET/CT images comparison before and after treatment (slices A–H) Panels A–H represent matched axial levels from the thoracic inlet to the cardiac upper border. The left two columns show baseline images obtained on June 5, 2025, including (1) contrast-enhanced CT and (2) corresponding positron emission tomography (PET/CT) fusion images. These baseline scans reveal a large, irregular anterior mediastinal mass with heterogeneous soft-tissue density and intense radiotracer uptake, consistent with aggressive metabolic activity of extensive-stage small-cell lung cancer. The right column shows follow-up CT images obtained on October 9, 2025, after five cycles of platinum–etoposide–atezolizumab therapy followed by consolidative radiotherapy. Across all slice levels (A–H), the previously identified tumor has nearly completely regressed, with restoration of normal mediastinal contours, resolution of paratracheal mass effect, and no residual measurable soft-tissue lesion. Lung aeration is markedly improved, and no new intrathoracic lesions are identified. This figure demonstrates a near-complete radiologic response both anatomically and metabolically, highlighting the patient’s exceptional treatment response.

As shown in the comparison image in Figure 4, Slices corresponding to the superior mediastinum, which initially demonstrated bulky nodal and soft-tissue involvement, showed complete disappearance of abnormal soft-tissue density and normalization of tracheal margins. Inferior slices involving the pericardial interface also demonstrated no residual mass-like lesion, and cardiac borders appeared fully restored. The disappearance of the structural correlate of the hypermetabolic tumor observed on baseline PET/CT suggests both anatomical and metabolic extinction of thoracic disease (Figure 4).

## Discussion

Interpretation in the context of existing evidence

The depth and rapidity of radiologic response observed in this patient are notable, given his age, comorbidities, and extensive metastatic burden. In pivotal trials such as IMpower133 (Clinical Trials.gov number, NCT02763579) [4] and CASPIAN (phase III CASPIAN study) [5], median overall survival for ES-SCLC treated with platinum-etoposide plus PD-L1 inhibitors was approximately 12-13 months, and complete or near-complete radiologic responses were rare [4,5]. Recent case reports have described exceptional responders achieving multi-year survival after platinum-etoposide-atezolizumab therapy, sometimes combined with thoracic radiotherapy, but such outcomes remain rare and uncommon, particularly in elderly patients with multi-organ involvement [13-15].

In the present case, the profound radiologic regression-approaching a near-complete response after only five cycles of therapy-raises the question of whether adjunctive metabolic modulation may have contributed to the observed outcome. High-dose vitamin C has been shown in preclinical and early clinical studies to generate tumor-selective oxidative stress, enhance DNA damage from chemotherapy and radiotherapy, and modulate antitumor immune responses. Nicotinamide has been implicated in the regulation of NAD+-dependent enzymes, DNA repair pathways, and epigenetic mechanisms, and a 2024 phase IIb trial in EGFR-mutant non-small cell lung cancer suggested that nicotinamide may enhance the efficacy of targeted therapy [12]. Although direct extrapolation to SCLC is not possible, these data support the biologic plausibility of nicotinamide as an adjunct to systemic anticancer treatment.

While causality cannot be established from a single case, it is conceivable that high-dose vitamin C and nicotinamide acted synergistically with chemoimmunotherapy and radiotherapy to potentiate antitumor effects in this patient. The integration of standard systemic therapy, consolidative radiation, and metabolic adjunctive supplementation may partially explain the unusually deep and rapid response observed. This case, therefore, serves as a hypothesis-generating observation that supports further investigation of metabolic adjunctive strategies in ES-SCLC. It should be acknowledged that RECIST-based response evaluation in this case was performed retrospectively and outside the context of a prospective clinical trial; therefore, definitive classification of complete response should be interpreted with caution.

## Conclusions

This case describes an elderly patient with extensive-stage small-cell lung cancer who achieved a near-complete thoracic radiologic remission after combined platinum-etoposide-atezolizumab therapy, consolidative thoracic and brain radiotherapy, and concurrent high-dose vitamin C and nicotinamide supplementation. Although definitive causal inference is not possible, the depth and rapidity of the response raise the possibility that adjunctive metabolic modulation may have contributed synergistically to treatment efficacy. These findings support further investigation into the potential role of high-dose vitamin supplementation, particularly vitamin C and nicotinamide, as complementary therapeutic strategies in conjunction with established chemoimmunotherapy for ES-SCLC.
